# *Mycobacterium tuberculosis *6-kDa Early Secreted Antigenic Target (ESAT-6) protein downregulates Lipopolysaccharide induced *c-myc *expression by modulating the Extracellular Signal Regulated Kinases 1/2

**DOI:** 10.1186/1471-2172-8-24

**Published:** 2007-10-03

**Authors:** Niladri Ganguly, Pham H Giang, Sandip K Basu, Fayaz Ahmad Mir, Imran Siddiqui, Pawan Sharma

**Affiliations:** 1 Immunology Group, International Centre for Genetic Engineering and Biotechnology Aruna Asaf Ali Marg, New Delhi-110067, India; 2 Department of Immunology, Max-Planck-Institute for Infection Biology, Chariteplatz 1, D-10117 Berlin, Germany

## Abstract

**Background:**

*Mycobacterium tuberculosis *(Mtb) causes death of 2–3 million people every year. The persistence of the pathogenic mycobacteria inside the macrophage occurs through modulation of host cell signaling which allows them, unlike the other non-pathogenic species, to survive inside the host. The secretory proteins of *M. tuberculosis *have gained attention in recent years both as vaccine candidates and diagnostic tools; they target the immune system and trigger a putatively protective response; however, they may also be involved in the clinical symptoms of the disease.

**Results:**

Our studies showed that RD-1-encoded secretory protein ESAT-6 is involved in modulation of the mitogen-activated protein (MAP) kinase-signaling pathway inside the macrophage. ESAT-6 induced phosphorylation of extracellular signal-regulated kinases 1/2 (ERK1/2) in the cytoplasm but not in the nucleus, which normally is the case for MAP kinases. ESAT-6 also antagonized LPS-induced ERK1/2 phosphorylation in the nucleus. Stimulation of cells by ESAT-6 along with sodium orthovanadate (a tyrosine phosphatase inhibitor) restored phosphorylation of ERK1/2 in the nucleus, suggesting active dephosphorylation of ERK1/2 by some putative phosphatase(s) in the nucleus. Further, ESAT-6 was found to down regulate the expression of LPS-inducible gene *c-myc *in an ERK1/2-dependent manner.

**Conclusion:**

This study showed the effect of secretory proteins of *M. tuberculosis *in the modulation of macrophage signaling pathways particularly ERK1/2 MAP kinase pathway. This modulation appears to be achieved by limiting the ERK1/2 activation in the nucleus which ultimately affects the macrophage gene expression. This could be a mechanism by which secretory proteins of Mtb might modulate gene expression inside the macrophages.

## Background

Tuberculosis, the disease caused by *Mycobacterium tuberculosis *(Mtb), is the leading cause of human mortality, claiming nearly 3 million lives every year [1]. The naïve or resting macrophages are extremely prone to invasion by Mtb bacilli and are unable to mount any anti-bacterial response associated with activated macrophages [2-7]. Thus, the resting macrophage seems to provide an ideal niche where intracellular tubercle bacilli may reside, replicate and persist [8,9]. The proteins that are secreted by mycobacteria have gained particular attention in the recent years both as vaccine candidates and virulence factors [10-18]. Some of these proteins like CFP-10 and ESAT-6 are encoded by the RD-1 region of Mtb genome, a region consistently deleted in all BCG vaccine strains of *M. bovis *[19-22].

Mitogen-activated protein kinases (MAPK) are evolutionarily conserved enzymes that are important in signal transduction. They play a diverse role in cell proliferation, cell death, cytokine production and cell differentiation. Three main families of MAPKs are found in mammalian cells: c-Jun-N-terminal kinases (JNK 1, 2 and 3); the extracellular signal-regulated kinases 1/2 (ERK1/2); and the p38 MAPK (p38 α, β, γ and δ) [23]. They play diverse roles in the cell, ranging from apoptosis, cell differentiation, cell proliferation, stress response, to production of proinflammatory cytokines etc. [24-31]. Targeting the MAP kinase pathway is one of the favorable strategies adopted by the pathogens to survive inside the macrophages [32]. Mycobacteria modulate MAPK signaling to promote their survival in the host cells. Studies on MAPKs have been done using virulent and attenuated strains of mycobacteria. *M. avium *has two strains; smooth transparent (SmT) and smooth opaque (SmO) which represent a more virulent and a less virulent phenotype, respectively. Both SmT and SmO induced early phosphorylation of p38 upon infection; however, only the attenuated strain elicited sustained activation of p38 MAPK. The virulent strains of mycobacteria caused greater inhibition of MAP kinases, particularly ERK1/2 pathway, as compared to the avirulent strains [33,34]. However, the molecular mechanisms involved in this phenomenon have not been investigated. Here, we show for the first time that ESAT-6 protein can modulate the ERK1/2 group of MAP kinases by limiting its activation in the nucleus. The MAP kinase-inducible transcription factor c-Myc is known to enhance cell proliferation as well as apoptosis [35,36]. Here we show that by modulating the MAP kinase ERK1/2, ESAT-6 down regulates the LPS-induced *c-myc *gene expression in the macrophages.

## Results

### ESAT-6 caused activation of extracellular signal regulated kinase1/2 (ERK1/2) in cytoplasm but not in nucleus

**Figure 1:**
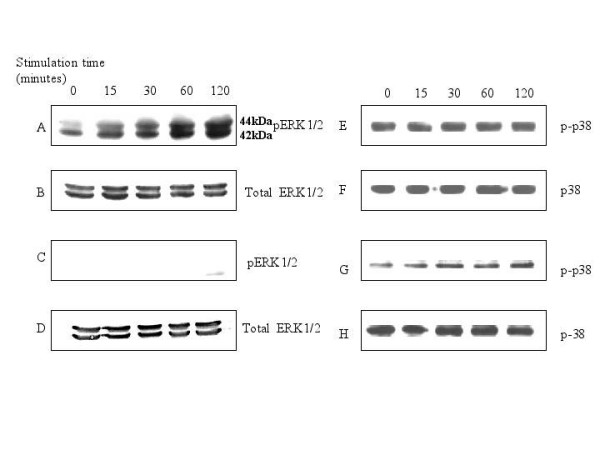
**ESAT-6 induced phosphorylation of ERK1/2 in cytoplasm but not in nucleus**. 10 × 10^6 ^RAW264.7 cells were stimulated with 5 μg/ml of recombinant ESAT-6 for 0, 15, 30, 60 and 120 minutes; cytoplasmic and nuclear extracts were run on gel and probed with anti-phospho-ERK1/2 antibody. (A) phosphorylation of ERK1/2 in cytoplasm. (C) phosphorylation of ERK1/2 in nucleus. (B) and (D) Total ERK1/2 in the cytoplasmic and nuclear extracts respectively at different time points to confirm equal loading of samples in all the lanes. (E) and (G) represents phosphorylated p38 in cytoplasm and the nucleus respectively. (F) and (H) shows the total p38 protein in cytoplasm and nucleus respectively. Data is a representative from three experiments.

We studied the effect of ESAT-6 on the activation status of ERK1/2 group of MAP kinases. MAP kinases are activated by a variety of extracellular stimuli such as stress, growth factors, and cytokines. The activation of ERK1/2 occurs through phosphorylation; the activated or phosphorylated ERK1/2 (pERK1/2) translocate to the nucleus [37] where they phosphorylate and activate the downstream cognate transcription factors such as CREB etc. [38]. We found that ESAT-6 (5 μg/ml) caused a time-dependent phosphorylation of ERK1/2 (Fig. 1A) in cytoplasm of RAW264.7 cells compared to unstimulated cells. In the Figure 1 the ERK1/2 is shown as a doublet where upper band represents ERK-1 with molecular weight of 44 kDa and the lower band represents ERK-2 with molecular weight of 42 kDa. In general cytoplasmic pERK1/2 would have translocated to the nucleus to activate the downstream molecules, but in the case of ESAT-6-stimulated cells we did not observe any pERK1/2 in the nuclear extract at any of the time points under the observation period (Fig. 1C). To determine whether the effect of ESAT-6 was specific for ERK1/2 or not, we checked for the phosphorylation of another MAP kinase p38. ESAT-6 triggered phosphorylation of p38 in both cytoplasm (Fig. 1E) and the nucleus (Fig. 1G), therefore the effect of ESAT-6 was specific for ERK1/2. Total p38 levels were constant over the experimental time period in both cytoplasm (Fig. 1F) and nucleus (Fig. 1H).

### Lipopolysaccharide triggered ERK1/2 phosphorylation in both cytoplasm and the nucleus

**Figure 2:**
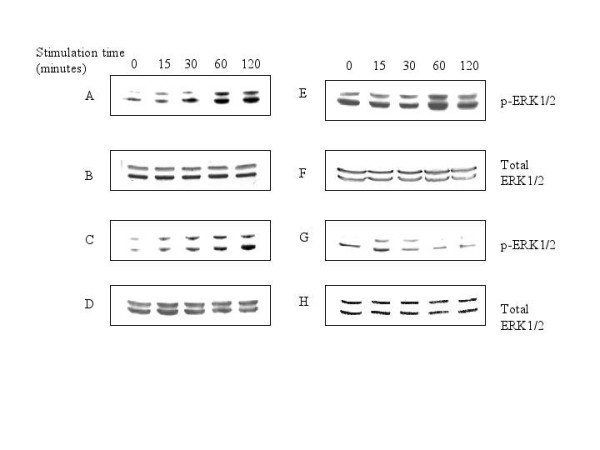
**LPS induced phosphorylation of ERK1/2 in both cytoplasm and nucleus**. RAW264.7 cells were stimulated with 0.1 μg/ml of bacterial LPS for 0, 15, 30, 60 and 120 minutes and probed for phospho-ERK1/2 as before. (A) Phosphorylation of ERK1/2 in cytoplasm upon stimulation with LPS(0.1 μg/ml). (C) phosphorylation of ERK1/2 in nucleus. The data is a representative of three independent experiments. (B) and (D) Total ERK1/2 in cytoplasm and nucleus normalized for protein content. Costimulation of RAW264.7 cells with LPS (0.1 μg/ml) and 5 μg/ml of ESAT-6 for 0, 15, 30, 60 and 120 minutes. (E) ERK1/2 phosphorylation in cytoplasm upon stimulation with 5 μg/ml of ESAT-6 and 0.1 μg/ml of LPS. (G) Phosphorylation of ERK1/2 in the nucleus with both LPS and ESAT-6. (F) and (H) Total ERK1/2 protein in cytoplasm and nucleus respectively.

The absence of pERK1/2 from the nucleus of ESAT-6-stimulated RAW264.7 cells was specific for ESAT-6 treatment. To establish this, we stimulated the cells with the bacterial lipopolysaccharide (LPS), which is a general activator of macrophages [39-47]. In the RAW264.7 cells stimulated with 0.1 μg/ml of LPS for the same time points as before, we observed time-dependent phosphorylation of ERK1/2 in both cytoplasm (Fig. 2A) and nucleus (Fig. 2C).

Next we wanted to know whether LPS can overcome the ESAT-6 imposed inhibition of phosphorylation of ERK1/2 in nucleus, for this RAW264.7 cells were co-stimulated for the same time points with LPS (0.1 μg/ml) and ESAT-6 (5 μg/ml). In the presence of ESAT-6, LPS caused only weak phosphorylation of ERK1/2 in nucleus (Fig. 2G) compared to the LPS alone. Thus, ESAT-6 seemed to dampen the ERK1/2 signaling of the MAP kinase family by limiting the activation of ERK1/2 in the nucleus.

### Diminished ERK1/2 activation in the nucleus by ESAT-6 was due to some tyrosine phosphatase

**Figure 3:**
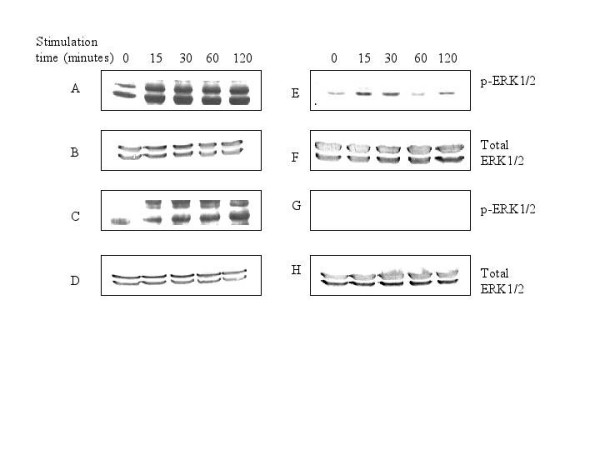
**Stimulation with ESAT-6 in presence of sodium orthovanadate caused appearance of phospho-ERK1/2 in the nucleus**. Stimulation of RAW264.7 cells with 5 μg/ml of ESAT-6 and 1 mM Na_3_VO_4 _for 0, 15, 30, 60 and 120 minutes. (A) Phosphorylation of ERK1/2 in cytoplasm. (C) ERK1/2 phosphorylation in the nucleus. (B) and (D) Total ERK1/2 in cytoplasm and nucleus respectively. (E) and (G) Phosphorylation of ERK1/2 in cytoplasm and the nucleus respectively upon treatment with 1 mM Na_3_VO_4 _for 0, 15, 30, 60 and 120 minutes. (F) and (H) Total ERK1/2 in cytoplasm and nucleus respectively to show equal loading of proteins in all the lanes. The data is representative of three independent experiments.

**Figure 4:**
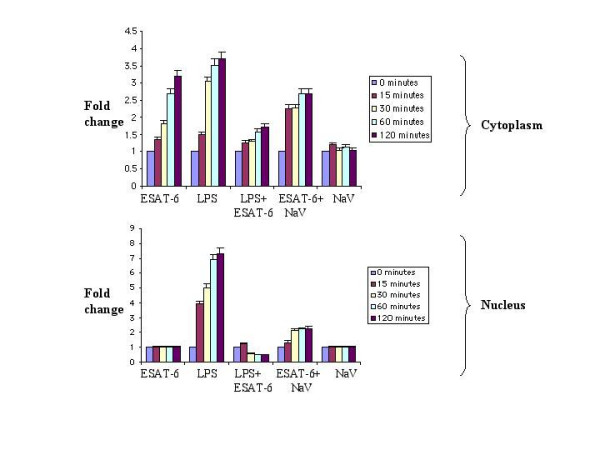
**Densitomteric analysis of the western blots**. The densitomteric analysis for the ERK blots for unstimulated cells, ESAT-6 and/or LPS, ESAT-6 and/or NaV are shown. The upper graph represents plot of cytoplasmic extracts and the lower graph represents nuclear extract. The data represented as fold change. The unstimulated cells were given a value of 1.00.

To evaluate whether any phosphatase was involved in the dephosphorylation of ERK1/2 in the nucleus of ESAT-6 treated cells, RAW264.7 cells were stimulated with 5 μg/ml of ESAT-6 in presence of 1 mM sodium orthovanadate (Na_3_VO_4_), which is a protein tyrosine phosphatase inhibitor [48]. We found that in the presence of Na_3_VO_4_, pERK1/2 appeared in the nucleus (Fig. 3C). The activation of ERK1/2 in cytoplasm was observed as usual (Fig. 3A). Since with ESAT-6 alone there was no pERK1/2 in the nucleus, but with Na_3_VO_4 _treatment there was phosphorylation of ERK1/2, so there might be some putative phosphatase(s) dephosphorylating the pERK1/2 as it translocated from cytoplasm to the nucleus. We checked whether sodium orthovanadate alone could induce activation of ERK1/2; in the cells stimulated with 1 mM Na_3_VO_4 _for the same time points, we found weakly activated ERK1/2 in cytoplasm (Fig. 3E) and none in the nucleus (Fig. 3G). The graphs showing densitomteric analysis of the above ERK blots are shown in Figure 4. The plots for cytoplasm and nucleus are shown separately.

**Figure 5:**
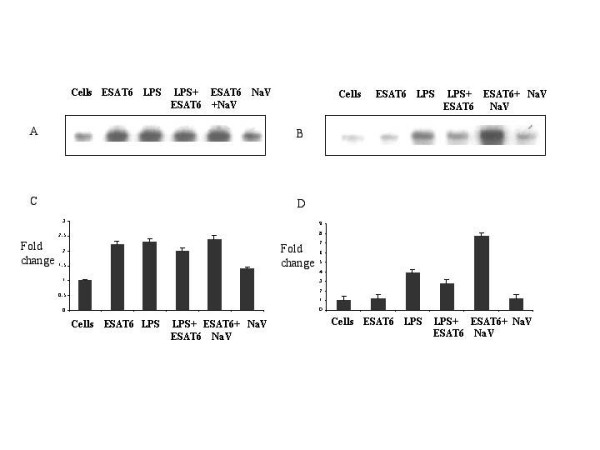
**ESAT-6 downregulated LPS-induced ERK kinase activity**. (A) and (B) represent the autoradiogram of the ERK kinase assay using Myelin basic protein (MBP) as a substrate in cytoplasm and nucleus respectively. Lane.1. Unstimulated cells, Lane.2. Cells stimulated with 5 μg/ml ESAT-6, Lane.3. Cells stimulated with 0.1 μg/ml of LPS, Lane.4. Cells stimulated with LPS and ESAT-6, Lane.5. Cells stimulated with 1 mM Na_3_VO_4 _and 5 μg/ml ESAT-6, Lane.6. Cells stimulated with 1 mM Na_3_VO_4_. (C) and (D) represents the graph showing fold change of the densitometric values obtained from the densitometric studies of the autoradiogram of (A) and (B) respectively. Unstimulated cells were given a value 1.00. The data represented as mean +/- S.D. of three independent experiments.

To further confirm the observations from western blotting, kinase assay for ERK1/2 was done. The RAW264.7 cells were treated with LPS and/or ESAT-6 and ESAT-6 and/or Na_3_VO_4 _for 60 minutes and the kinase activity was assayed as described in Methods. In cytoplasm (Fig. 5A) both LPS and ESAT-6 increased ERK enzyme activity over basal level. ESAT-6 treatment was found to have no effect on the ERK kinase activity in the nucleus over the basal level (Fig 5B); furthermore, ESAT-6 antagonized the LPS-induced ERK activation. Concurrent treatment with Na_3_VO_4 _and ESAT-6 increased ERK activation in the nucleus by more than 4-fold compared to the ESAT-6 alone (Fig. 5D). Na_3_VO_4 _alone did not have any effect on ERK kinase activity over the basal level. Thus the kinase assay confirmed the earlier western blot observations.

### ESAT-6 stimulated phosphatase activity in the nucleus

**Figure 6:**
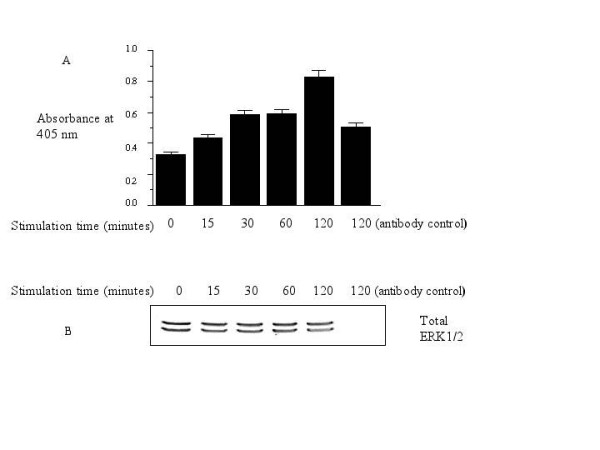
**ESAT-6 stimulated increase in the phosphatase activity associated with ERK1/2 in the nucleus**. (A) RAW264.7 cells were stimulated for different time points of 0, 15, 30, 60 and 120 minutes, the ERK-1 was immunoprecipitated from the nuclear extract and the phosphatase activity was determined, the last column where cells were stimulated with ESAT-6 for 120 minutes but no ERK-1 antibody was added (antibody control). (B) After phosphatase assay was done, the immunoprecipitate was mixed with 2× sample buffer and run on 10% SDS-PAGE and after western blotting the membrane was probed with ERK-1 antibody to confirm equal pull down of ERK1/2 in all the samples. The graph shows the mean +/- S.D. of three independent experiments.

In order to ascertain if the absence of pERK1/2 in nucleus was really due to some phosphatase(s), we determined phosphatase activity associated with ERK1/2 for the same time points in the nucleus. Determination of phosphatase activity showed that upon stimulation with ESAT-6 there was 1.5 fold increase in the phosphatase activity at 15 minutes, and 2.5 fold at 120 minutes (Fig. 6A); the antibody control (last column) in which cells were stimulated with ESAT-6 for 120 minutes but were not treated with anti-ERK-1 antibody, showed less than 1.5 fold increase in phosphatase activity over the basal level. The total ERK1/2 protein levels were found to be uniform in all the immunoprecipitated samples and absent in the antibody control (Fig. 6B).

### ESAT-6 downregulated LPS induced *c-myc *expression

**Figure 7:**
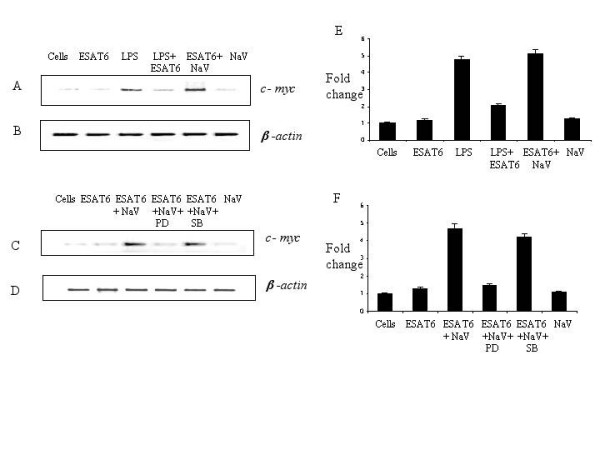
**ESAT-6 downregulated LPS induced *c-myc *expression**. (A) RT-PCR for *c-myc *gene expression, Lane. 1. Unstimulated cells, Lane.2. Cells stimulated with 5 μg/ml ESAT-6, Lane.3. Cells stimulated with 0.1 μg/ml of LPS, Lane. 4. Cells stimulated with LPS and ESAT-6, Lane.5. Cells stimulated with 1 mM Na_3_VO_4 _and 5 μg/ml ESAT-6, Lane.6. Cells stimulated with 1 mM Na_3_VO_4_. Stimulation time was 120 minutes. (C) RT-PCR for *c-myc *gene expression, Lane.1. Unstimulated cells, Lane.2. Cells stimulated with 5 μg/ml ESAT-6, Lane.3. Cells stimulated with 1 mM Na_3_VO_4 _and 5 μg/ml ESAT-6, Lane.4. Cells stimulated with Na_3_VO_4 _and ESAT-6 and 10 μM of MEK-1 inhibitor PD98059, Lane.5. Cells simulated with Na_3_VO_4 _and ESAT-6 and 10 μM of p38 inhibitor SB203580, Lane.6. Cells stimulated with 1 mM Na_3_VO_4_, (B) and (D) RT-PCR for β-*actin *to confirm equal amplification in all the samples. The data is representative of two independent experiments. (E) and (F) shows the graph of densitometric analysis of Figure. 8A and 8C respectively. The data is shown as fold change; unstimulated cells were given a value of 1.00.

Next, we looked whether ESAT-6 could exert any effect at the level of gene expression; we monitored the expression of *c-myc *encoding a transcription factor c-Myc, which is regulated by MAP kinases [24,49-52]. The protein c-Myc plays a role in cell proliferation and programmed cell death [35,36]. We monitored the *c-myc *mRNA levels by RT-PCR at 120 minutes after the addition of different stimuli. We found that ESAT-6 did not change the expression of *c-myc *over the basal level; however, the LPS (0.1 μg/ml)-induced *c-myc *expression was downregulated by ESAT-6. Also, addition of 1 mM Na_3_VO_4 _along with ESAT-6 increased the level of *c-myc *expression compared to that obtained with ESAT-6 alone while Na_3_VO_4 _alone did not affect the basal expression level of *c-myc *(Fig. 7A).

**Figure 8:**
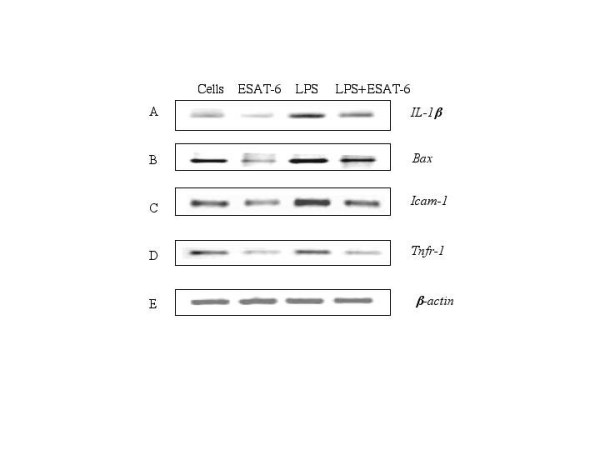
**ESAT-6 downregulated LPS-induced expression of several genes**. (A-E) RT-PCR for the genes *IL-1β*, *Bax*, *Icam-1*, *Tnfr-1 *and β-*actin*. The data is representative of two independent experiments. Lane.1. Unstimulated cells, Lane.2. Cells stimulated with 5 μg/ml ESAT-6, Lane.3. Cells stimulated with 0.1 μg/ml of LPS, Lane.4. Cells stimulated with LPS and ESAT-6. (F) RT-PCR for β-*actin *to confirm equal amplification in all the samples.

To determine whether the downregulation of *c-myc *gene expression by ESAT-6 was a consequence of the inhibited activation of ERK1/2 in the nucleus, we incubated the cells with ESAT-6 and Na_3_VO_4 _along with MEK-1 inhibitor PD98059 (10 μM) [53,54] and also p38 MAP kinase inhibitor SB203580 (10 μM) [55,56]. As observed before, treatment with Na_3_VO_4 _and ESAT-6 enhanced the *c-myc *expression over ESAT-6 stimulation. Interestingly treatment with MEK-1 inhibitor PD98059 downregulated the *c-myc *expression to the level obtained with ESAT-6 stimulation while the p38 inhibitor SB203580 had no effect on *c-myc *expression levels (Fig. 7C). Since the addition of SB203580 did not have any effect on *c-myc *levels, the p38 MAP kinase pathway was not involved in *c-myc *expression. Additionally, we looked at the effect of ESAT-6 on the expression on LPS-inducible genes. ESAT-6 was also found to down regulate LPS-induction of several genes *IL-1β*, *Bax*, *Icam-1*, and *Tnfr-1 *(Fig. 8).

## Discussion

The present study demonstrates that ESAT-6 modulated the ERK1/2 group of MAP kinase. We also found that this modulation was achieved by inhibition of phosphorylation of ERK1/2 in the nucleus. However, the phosphorylation of another MAP kinase p38 was not affected by ESAT-6. Nevertheless, LPS, a general macrophage activator [39-47] triggered phosphorylation of ERK1/2 in both cytoplasm and the nucleus. This showed that the limited activation of ERK1/2 in the nucleus was specific for ESAT-6 stimulation. Costimulation of cells with LPS and ESAT-6 dampened the ERK1/2 phosphorylation in the nucleus compared to that obtained with LPS alone; clearly ESAT-6 was exerting a strong inhibitory effect on the phosphorylation of ERK1/2 in the nucleus. Our finding that the treatment of cells with Na_3_VO_4_, a tyrosine phosphatase inhibitor [48] along with ESAT-6 caused pERK1/2 to appear in the nucleus indicated that there was some phosphatase(s) activity in the nucleus that was triggered upon stimulation with ESAT-6. Moreover, when this phosphatase activity was suppressed by Na_3_VO_4_, the pERK1/2 reappeared in the nucleus. The results of kinase assay further corroborated our observations from western blotting that phosphorylation of ERK1/2 was concomitant with its activation. Measurement of phosphatase activity associated with ERK1/2 in the nucleus showed that there was an increase in this activity over the given time period; this finding was consistent with our observation that following treatment with both ESAT-6 and a phosphatase inhibitor (Na_3_VO_4_) there was an increase in phosphorylation of ERK1/2. It was already established that ERK1/2 after getting phosphorylated in cytoplasm translocates to the nucleus [37]; therefore at zero minute, we observed little pERK1/2 in the nucleus (Fig. 1C). Our findings tend to suggest that although with increase in the ERK1/2 phosphorylation in the cytoplasm of the ESAT-6-stimulated cells, pERK1/2 must have migrated to the nucleus, but increasing phosphatase activity in the nucleus, again associated with ESAT-6 stimulation, dephosphorylated the pERK1/2 coming from the cytoplasm; therefore no pERK1/2 was detectable in the nuclear extract. Since MAP kinases undergo rapid turnover in the nucleus, the levels of total ERK1/2 in the nucleus remained constant over the experimental time period.

The *c-myc *is one of the early response genes that encode a transcription factor c-Myc, which is a key regulator of cell proliferation and apoptosis. Since *c-myc *expression was reported to occur through Ras/Raf/MEK/ERK pathway [24,49-52], we studied the effect of ESAT-6 on *c-myc *expression in RAW264.7 cells. ESAT-6 itself did not have any effect on *c-myc *expression over the basal level. However the LPS induced *c-myc *expression was found to be downregulated by ESAT-6 compared to LPS stimulation alone. Again treatment with ESAT-6 along with 1 mM Na_3_VO_4 _increased the level of *c-myc *compared to that observed with ESAT-6 alone while Na_3_VO_4 _alone did not have any effect on *c-myc *levels. These results can be explained by the dampening of LPS-induced ERK1/2 phosphorylation in the nucleus by ESAT-6. As noted above, treatment with Na_3_VO_4 _along with ESAT-6 resulted in an increased level of ERK1/2 activation in the nucleus compared to ESAT-6 alone. This differential activation of ERK1/2 pathway resulted in differential *c-myc *expression. To further confirm the role of ERK1/2 pathway in *c-myc *expression, we determined *c-myc *expression in the presence of MEK-1 inhibitor PD98059 [53,54] and p38 MAP kinase inhibitor SB203580 [55,56] along with Na_3_VO_4 _and ESAT-6. PD98059 downregulated *c-myc *levels while SB203580 did not have any effect on *c-myc *levels. The activation of ERK1/2 pathway in nucleus upon treatment with Na_3_VO_4 _and ESAT-6 was abrogated by PD98059 and hence *c-myc *levels were downregulated. Since SB203580 did not have any effect on *c-myc *expression, p38 MAP kinase was not involved in the gene expression. It confirmed the earlier observations of p38 phosphorylation from western blotting where there was no inhibition in p38 activation in cytoplasm or nucleus by ESAT-6.

Although there are reports that CFP-10 forms a 1:1 complex with ESAT-6 [57]; however other studies [58] have shown that there is discordance between secretion of CFP-10 and ESAT-6. Okkels and colleagues [59] have shown that there are as many as 8 different forms of ESAT-6 and that the acetylation of ESAT-6 was required for complexation with CFP-10. Another study has shown that ESAT-6 as well as the CFP-10:ESAT-6 complex inhibited the PI-3 kinase-Akt signaling, indicating that the active component involved in downregulating the macrophage signaling was the ESAT-6 [60]. Our studies with CFP-10 and CFP-10:ESAT-6 complex did not show any inhibition of the ERK1/2 phosphorylation in cytoplasm or nucleus of the RAW264.7 cells (see Additional file 1). It has also been shown that ESAT-6 binds to the Toll-like receptor-2 (TLR-2) and not TLR-4 on the surface of RAW264.7 macrophages, and causes inhibition of activation of transcription factors NF-κB and Interferon regulatory factors (IRFs) through the Akt kinase pathway [60]. Our studies suggest yet another mechanism, *viz*., modulation of the ERK arm of the MAP kinase pathway, by which ESAT-6 could bring about deactivation of the host cell.

## Conclusion

This study has shown that mycobacterial secretory protein ESAT-6 could inhibit ERK1/2 activation in the nucleus of RAW264.7 cells. This inhibition resulted in downregulation of LPS-induced ERK1/2 activation in the nucleus and subsequent expression of c-Myc, a key factor in macrophage activation. These findings underline the role of ESAT-6 in deactivation of the macrophage, the host cell for *M. tuberculosis*.

## Methods

### Reagents and Antibodies

Bacterial lipopolysaccharide (LPS) and p-nitro phenylphosphate (p-NPP) and other fine chemicals were obtained from Sigma, St. Louis, MO, USA. Antibodies against ERK-1 and phospho-ERK1/2 were obtained from Santa Cruz Biotech, CA, USA. Tissue culture medium RPMI-1640 and the antibiotics penicillin and streptomycin and fetal bovine serum were from Life Technologies, USA.

### Maintenance of cell line

Murine macrophage cell line RAW264.7 transformed with Abelson murine leukemia virus, originally obtained from ATCC, was routinely maintained in RPMI-1640 medium containing 2 mM glutamine, 100 μg/ml of penicillin and streptomycin and 10% fetal bovine serum at 5% CO_2 _in a humidified atmosphere at 37°C.

### Cloning, expression and purification of recombinant Mycobacterial (Mtb) ESAT-6 protein

The open reading frame *Rv3875*, encoding ESAT-6 (GenBank Accession no. AF420491) of *M. tuberculosis*, was amplified by PCR from the genomic DNA of a local clinical isolate, by using the following primers: forward, 5'-GGAATTCCATATGACAGAGCAGCAGTGGAATTTCG-3', reverse, 5'-CCGCTCGAGTGCGAACATCCCAGTGACGTTGC-3' (*Nde*I and *Xho*I sites, respectively, are underlined). The PCR product obtained here was cloned in the pGEM-T-Easy^® ^vector and the nucleotide sequence of the gene revalidated. Full-length authentic gene was then sub-cloned into bacterial expression vector pET23b+; this vector yielded satisfactory levels of polyhistdine-tagged recombinant ESAT-6 protein expressed as an insoluble protein in *E. coli*. From the inclusion bodies, the protein was extracted using 8 M Urea pH 8.0. Recombinant ESAT-6 was purified by nickel-nitrilotriacetic acid (Ni^2+^-NTA) metal affinity chromatography according to the manufacturer's recommendations for purification of proteins under denaturing conditions. After purification, the pure fractions of protein were pooled together and the urea was removed by dialysing against 10 mM Na_2_HPO_4_, pH 8.0. The dialysed protein was aliquoted and kept at -20°C. The endotoxin level in the protein did not exceed 0.03 endotoxin units as done by E-toxate kit (Sigma).

### Western blot analysis

For western blotting, 10 × 10^6 ^RAW264.7 cells were seeded per well of 12-well tissue culture plate in 1 ml of RPMI-1640 medium containing 10% FBS; cells were stimulated with 5 μg/ml of recombinant ESAT-6 for 0, 15, 30, 60 and 120 minutes. After stimulation, cells were harvested and lysed in 300 μl of lysis buffer (10 mM HEPES pH 7.9, 10 mM KCl, 0.1 mM EDTA, 0.1 mM EGTA, 1 mM PMSF, 1 mM sodium orthovanadate (Na_3_VO_4_), 1 mM sodium fluoride, 1 μg/ml each of Leupeptin, Pepstatin A and Aprotinin, and 1% NP-40) for 20 minutes on ice. The cell lysates so obtained were cleared by centrifugation at 13,000 rpm, the supernatant represented the cytoplasmic extract; the nuclear pellet was washed and resuspended in the nuclear extraction buffer (20 mM HEPES pH 7.9, 400 mM KCl, 10 mM EDTA, 10 mM EGTA), kept on ice for 40 minutes with intermittent vortexing. Finally, the suspension was centrifuged at 13,000 rpm at 4°C, the supernatant was the nuclear extract. The protein contents of the cytoplasmic as well as nuclear extracts were estimated by the Bradford method and was then run on gel.

### Phosphatase assay

For determination of phosphatase activity, 40 × 10^6 ^RAW264.7 cells were plated per well in a 6-well tissue culture plate (Nunc, Roskilde, Denmark) in 2 ml of complete medium. Cells were stimulated with 5 μg/ml of ESAT-6 for 0, 15, 30, 60 and 120 minutes. After stimulation, cells were harvested and lysed in 2 ml of lysis buffer for 20 minutes at 4°C, then the suspension was centrifuged at 13,000 rpm and the supernatant was discarded; the nuclear pellet was washed and suspended in 300 μl of nuclear extraction buffer and kept on ice for 40 minutes with intermittent vortexing. Then the suspension was centrifuged at 13,000 rpm and the supernatant was 'nuclear extract'. To the nuclear extract so prepared was added 20 μl of 30% ProteinA-agarose, and kept on nutator for 1 hour at 4°C (pre-clearing); to the cleared supernatant was added 4 μl of anti-ERK-1 antibody and kept on nutator for 1.5 hours at 4°C, followed by addition of 40 μl of 30% ProteinA-agarose; this mixture was kept on nutator for another 1 hour, then the Protein A-agarose beads carrying immunoprecipitated ERK were pelleted at 2,000 rpm; the pellet (immunoprecipitate) was washed thrice with wash buffer (50 mM HEPES pH 7.5, 2.5 mM MgCl_2_, 5% glycerol and 0.05% TritonX-100), and suspended in 100 μl of substrate solution (1 mg of p-nitrophenyl phosphate in 1 ml of buffer containing 50 mM MES pH 6.0, 1 mM EDTA and 0.1% Triton X-100) and kept at 37°C for 30 minutes. Then the agarose beads were pelleted at 2,000 rpm and the supernatant from each reaction tube was dispensed, 100 μl/well, into a 96-well micro-ELISA plate; to each such well 5 μl of 10 N NaOH to stop the reaction and the absorbance of resultant yellow color read at 405 nm using a microplate reader.

### Kinase Assay for ERK1/2

For ERK1/2 kinase assay, ERK1/2 was immunoprecipitated from untreated and LPS and/or ESAT-6 treated RAW264.7 cells (2 × 10^7^/treatment) for 60 minutes. Then cells were lysed and cytoplasmic and nuclear extracts were prepared. From the extracts, ERK was immunoprecipitated using anti-ERK-1 antibody. The immunoprecipitates were washed with wash buffer (20 mM Tris-HCl (pH 7.5), 20 mM MgCl_2_, 2 mM DTT, 1 mM pNPP and 10 μM sodium orthovanadate) and then incubated with 20 μl of kinase reaction buffer (20 mM Tris-HCl pH7.5, 20 mM MgCl_2_, 2 mM DTT, 10 μM ATP, 10 μCi γ-^32^P-ATP and 5 μg MBP). The reaction was carried out at 30°C for 10 minutes. The reaction was terminated by addition of equal volume of 2× SDS loading buffer followed by boiling for 5 min. The reaction mixtures were subjected to SDS polyacrylamide gel electrophoresis. Dried gels were then exposed to X-ray films and the amount of [^32^P]-ATP incorporation in the substrate were ascertained by autoradiography followed by densitometric analysis.

### Reverse transcription-PCR

Total RNA was isolated from 10 × 10^6 ^RAW264.7 cells, using 1 ml of TriZOL Reagent (Invitrogen Inc., Carlsbad, CA, USA); the total RNA was then quantified and converted to cDNA using Superscript II reverse transcriptase (Invitrogen Inc., USA). The cDNA was then used for amplification by PCR. The PCR was done using the Taq DNA polynerase (Biotools, B&M Lab, S.A., Spain). The PCR conditions were as follows: 94°C – 5 minutes (hot start), 94°C – 1 minute (denaturation), 55°C – 1 minute (annealing), 72°C – 1 minute (extension), 72°C – 10 minutes (final extension). The primers for amplification of *c-myc*: Forward: 5'-TCC TGT ACC TCG TCC GAT TC-3', Reverse: 5'-AAT TCA GGG ATC TGG TCA CG-3', *IL-1β*: Forward: 5'-TGG CAA CTG TTC CTG AAC TCA A-3', Reverse: 5'-TCC ACG GGA AAG ACA CAG GTA-3', *Icam-1*: Forward: 5'-TCT CGG AAG GGA GCC AAG TAA-3', Reverse: 5'-CTC TTG CCA GGT CCA GTT CC-3', *Tnfr-1*: Forward: 5'-CCC CAC CTC TGT TCA GAA ATG G-3', Reverse: 5'-TAC TTC CAG CGT GTC CTC GT-3', *Bax*: Forward: 5'-CTG AGC TGA CCT TGG AGC AG-3', Reverse: 5'-CCA GCC CAT GAT GGT TCT GAT-3', β-*actin*: Forward: 5'-CTA TGC TCT CCC TCA CGC CA-3', Reverse: 5'-CCG CTC GTT GCC AAT AGT GAT-3'.

## Abbreviations

ATCC – American Type Culture Collection, ERK1/2 – Extracellular Signal Regulated Kinases1/2, ESAT-6 – Early Secreted Antigenic Target 6-kDa, LPS – Lipopolysaccharide, MBP – Myelin Basic Protein, Mtb – *Mycobacterium tuberculosis*, MAP kinase – Mitogen Activated Protein kinase, pERK1/2 – phosphoERK1/2, p-NPP – *para*-nitrophenyl phosphate.

## Authors' contributions

NG performed the western blot analysis, kinase assay and phosphatase assay. SKB and IS did the RT-PCR experiments. PHG and FAM helped in the expression and purification of recombinant ESAT-6 and in the western blot analysis. PS and NG were responsible for conceptualizing and designing the study as well as for writing the manuscript. All authors have read and approved the final manuscript.

## Supplementary Material

Additional file 1 Western blot analysis of ERK1/2 phosphorylation upon stimulation by CFP-10 and CFP10:ESAT6 complex. The data shows the phosphorylation of ERK1/2 in cytoplasmic and nuclear extracts upon stimulation with CFP-10 and CFP10:ESAT6 complex for 0, 15, 30, 60 and 120 minutes. Click here for file
