# Supplementary material for: Mycobacterium tuberculosis 6-kDa Early Secreted Antigenic Target (ESAT-6) protein downregulates Lipopolysaccharide induced c-myc expression by modulating the Extracellular Signal Regulated Kinases 1/2
Source: BMC Immunol. 2007 Oct 3;8:24. doi: 10.1186/1471-2172-8-24 (PMC2082026; doi:10.1186/1471-2172-8-24)
Supplement: Additional file 1 — Western blot analysis of ERK1/2 phosphorylation upon stimulation by CFP-10 and CFP10:ESAT6 complex. The data shows the phosphorylation of ERK1/2 in cytoplasmic and nuclear extracts upon stimulation with CFP-10 and CFP10:ESAT6 complex for 0, 15, 30, 60 and 120 minutes. [file 1471-2172-8-24-S1.pdf]

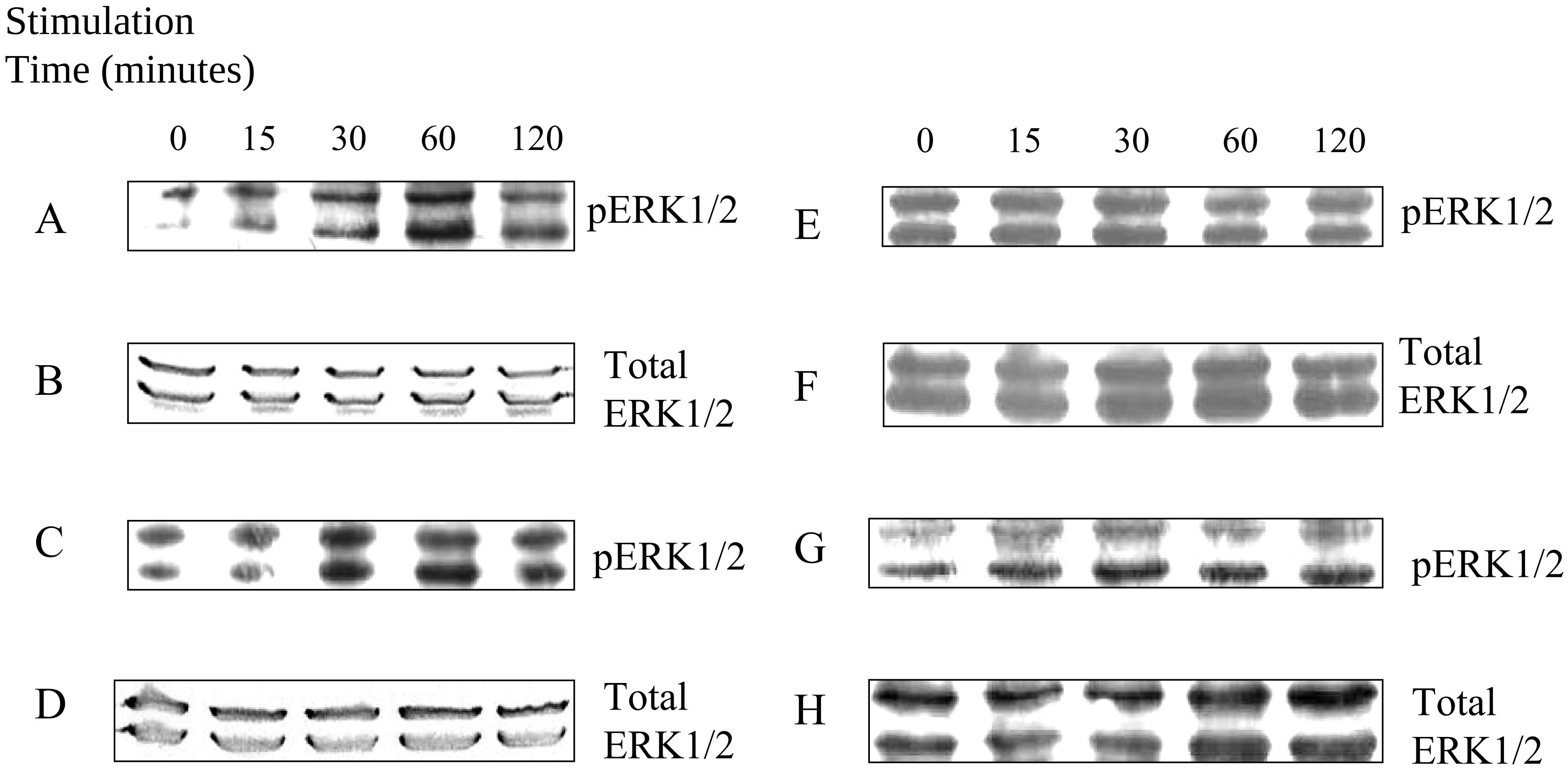
Stimulation
Time (minutes)
0
15
30
60
120
A
pERK1/2
B
Total
ERK1/2
C
pERK1/2
D
Total
ERK1/2
0
15
30
60
120
E
pERK1/2
F
Total
ERK1/2
G
pERK1/2
H
Total
ERK1/2

Figure Legends

Figure.S1

CFP-10 induced ERK1/2 phosphorylation in both cytoplasm and nucleus. $10x10^6$ RAW264.7 cells were stimulated with 5μg/ml of recombinant CFP-10 for 0, 15, 30, 60 and 120 minutes; cytoplasmic and nuclear extracts were run on gel and probed with anti-phospho-ERK1/2 antibody. (A) phosphorylation of ERK1/2 in cytoplasm. (C) phosphorylation of ERK1/2 in nucleus. (B) and (D) Total ERK1/2 in the cytoplasmic and nuclear extracts respectively at different time points to confirm equal loading of samples in all the lanes.

Figure.S2

CFP10:ESAT6 complex induced ERK1/2 phosphorylation in both cytoplasm and nucleus. 10×10^6 RAW264.7 cells were stimulated with 5µg/ml of recombinant CFP10:ESAT6 complex for 0, 15, 30, 60 and 120 minutes; cytoplasmic and nuclear extracts were run on gel and probed with anti-phospho-ERK1/2 antibody. (E) phosphorylation of ERK1/2 in cytoplasm. (G) phosphorylation of ERK1/2 in nucleus. (F) and (H) Total ERK1/2 in the cytoplasmic and nuclear extracts respectively at different time points to confirm equal loading of samples in all the lanes.
